# Posterior Reversible Encephalopathy Syndrome Associated With Anlotinib: A Case Report and Literature Review

**DOI:** 10.3389/fneur.2021.546481

**Published:** 2021-05-06

**Authors:** Di Nan, Xiang Yin, Di Ma, Xiaoyu Jiang, Baihua Wu, Jiachun Feng

**Affiliations:** Department of Neurology and Neuroscience Center, The First Hospital of Jilin University, Changchun, China

**Keywords:** posterior reversible encephalopathy syndrome, anlotinib, adverse effects, targeted therapy, case report

## Abstract

Posterior reversible encephalopathy syndrome (PRES) is a relatively rare clinical disease, characterized by reversible subcortical vasogenic edema. Here, we present the first reported case of PRES induced by anlotinib, a multi-target tyrosine kinase inhibitor. A 56-year-old female patient with lung adenocarcinoma and bone metastasis experienced hypertension and mental confusion when she received anti-angiogenesis treatment. PRES was diagnosed after magnetic resonance of the patient's brain revealed hyperintensities bilaterally around the cerebellum, pons, fronto-parieto-occipital areas, and corona radiate. Diffusion-weighted imaging showed hyperintensities bilaterally in the parieto-occipital cortical regions. Subsequently, the patient was diagnosed with PRES, and remission was achieved with anti-hypertensive drugs. Six cases of rare adverse effects induced by anlotinib were reviewed in the literature. Since anlotinib has been widely applied as a novel third-line treatment in patients with non-small-cell lung cancer, the association between PRES and anlotinib would benefit neurologists and oncologists in future diagnoses and treatment.

## Background

Posterior reversible encephalopathy syndrome (PRES) was first reported by Hinchey et al. (1) in 1996 and is characterized by seizures, encephalopathy, confusion, headaches, and visual disturbances. Moreover, patients with severe hypertension, fluctuations in blood pressure, renal failure, immunosuppressant therapy, chemotherapy, eclampsia, and autoimmune disorder have an increased risk of developing this condition (2).

Anlotinib is a tyrosine kinase inhibitor inhibiting angiogenesis targeting vascular endothelial growth factor receptor (VEGF), fibroblast growth factor receptor, platelet-derived growth factor receptor, c-Kit, and c-MET (3). It has been approved by the China National Medical Products Administration (NMPA) in 2018 and has been widely used to treat patients with advanced non-small-cell lung cancer (NSCLC) who have undergone at least two lines of chemotherapy. A study reporting the phase I clinical trial outcomes for this drug found that the safety dosage is 12 mg/day (4) and that the most common adverse effects related to nervous system disorder was reported to be dizziness and headache in the ALTER0303 trial (5). In addition, there are a few rare adverse effects reported (6–10). Though there have been cases of PRES induced by other VEGF inhibitors, anlotinib has not been previously reported to be associated with PRES (11–13). Here, we present the case of a 56-year-old female patient with lung adenocarcinoma and bone metastasis who developed PRES after being treated with anlotinib.

## Case Presentation

Five-years prior to the hospitalization for PRES, the patient was diagnosed with peripheral lung cancer and bone metastasis in 2013. Percutaneous puncture biopsy supported the diagnosis of adenocarcinoma and the patient was subsequently treated with icotinib for 3 years; however, it failed to limit the progression of the disease. Following this, her oncologists discontinued icotinib and switched to a combined therapy of Taxol plus platinum-based chemotherapy and Pemetrexed plus platinum-based chemotherapy in order to prevent the rapid progression of her cancer. After this course of treatment, she underwent a percutaneous puncture biopsy and genetic testing for EGFR and T90M mutations, which tested negative. Magnetic resonance imaging (MRI) of her brain was also performed and it did not reveal any abnormalities. In August 2018, the patient who had no history of hypertension began anlotinib treatment (12 mg/day) and developed several side-effects as a result of this treatment including poor appetite, fluctuating blood pressure, and headaches which persisted. However, her treating physician did not find any neurological deficits prior to her hospitalization in 2019.

**Figure 1:**
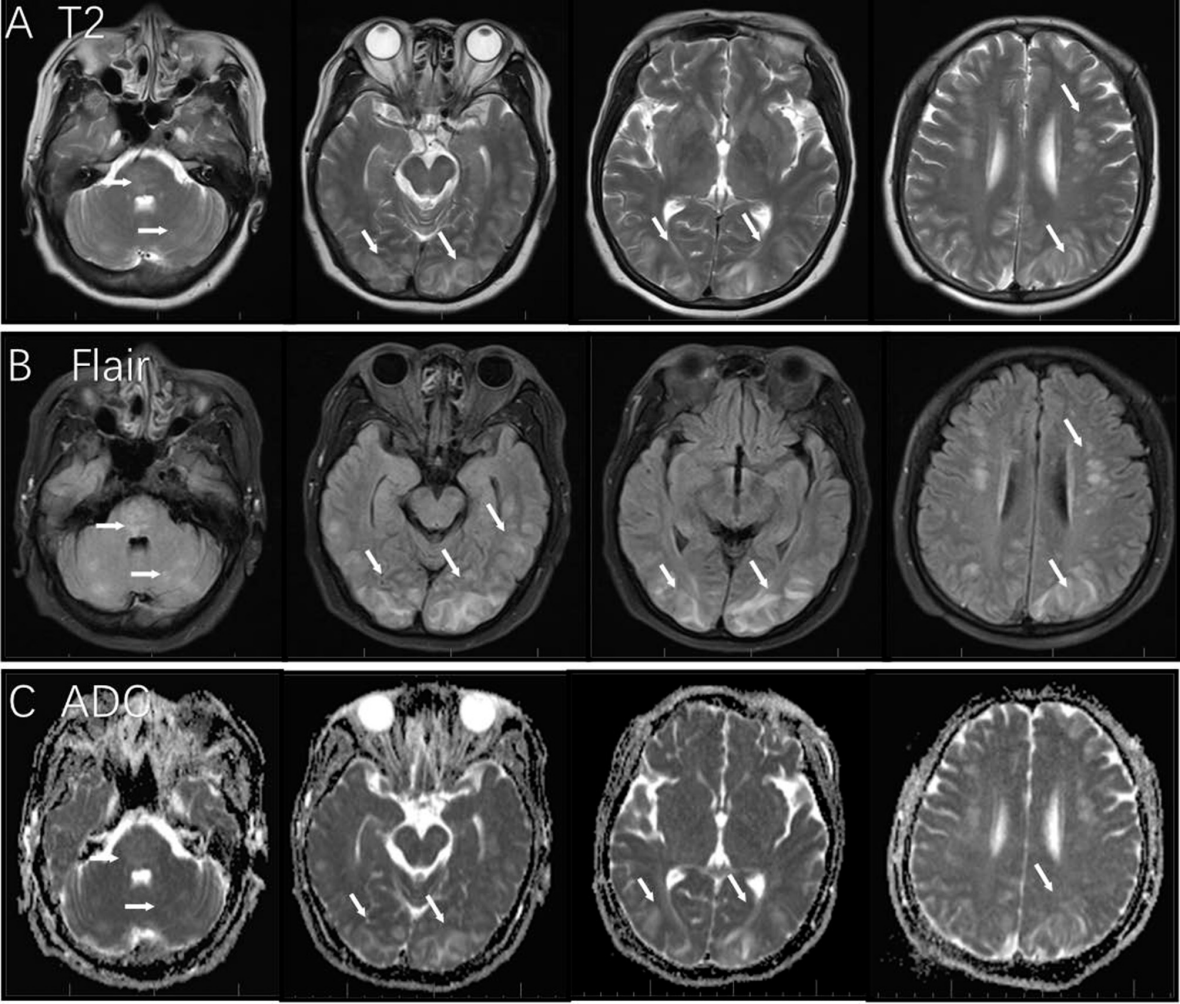
MRI findings in this case of posterior reversible encephalopathy syndrome. T2 and Flair image showed hyperintensity in the area of the cerebellum, pons, fronto-parieto-occipital areas, and corona radiate [white arrows in panels **(A,B)**]; bilateral parieto-occipital cortical regions showed hyperintensity in ADC [white arrows in panel **(C)**].

On February 2019, the 56-year-old female patient was admitted to emergency department after complaining of headaches and vomiting. On admittance, she presented with elevated blood pressure (217/120 mmHg) which had reportedly persisted for 7 days prior to admission. She also presented with mental confusion which progressed over the course of the day. Upon neurological examination, her speech was unclear. She presented with normal muscle strength in her extremities, her reflexes were normal. A routine blood test including renal function, liver function, D-dimer, and ion were unremarkable. However, her C-reactive protein (CRP) level was slightly elevated. A computed tomography (CT) scan of her brain showed hypodense signals bilaterally in her subcortical and cortical occipital regions. Gadopentetate dimeglumine (Gd)-enhanced MRI identified T2 and fluid-attenuated inversion recovery (FLAIR) hyperintensities as well as T1 hypointensities bilaterally in the cerebellum, pons, fronto-parieto-occipital areas, and corona radiate. Apparent Diffusion Coefficient (ADC) showed hyperintensities bilaterally in the parieto-occipital cortical regions (Figure 1). No enhancement was observed.

The first day after admission, a diagnosis of PRES was made based on the patient's clinical presentation, her recent patient history, and by the results of the imaging studies. In response to this diagnosis, anlotinib was discontinued on the recommendation of the consulting oncologists and the patient received intravenous urapidil and mannitol to reduce her blood pressure and prevent vasogenic edemas. Her blood pressure was maintained at 130–140/80–90 mmHg and her neurological symptoms significantly improved after 5 days. However, the patient appeared to show signs of distress from her medical episode and as a result, she refused to undergo any follow-up imaging and she decided to prematurely end her stay in the hospital. The patient was prescribed a treatment course of Nifedipine GITS (30 mg/day) and daily monitoring of blood pressure. No further oncological treatments were administered.

**Figure 2:**
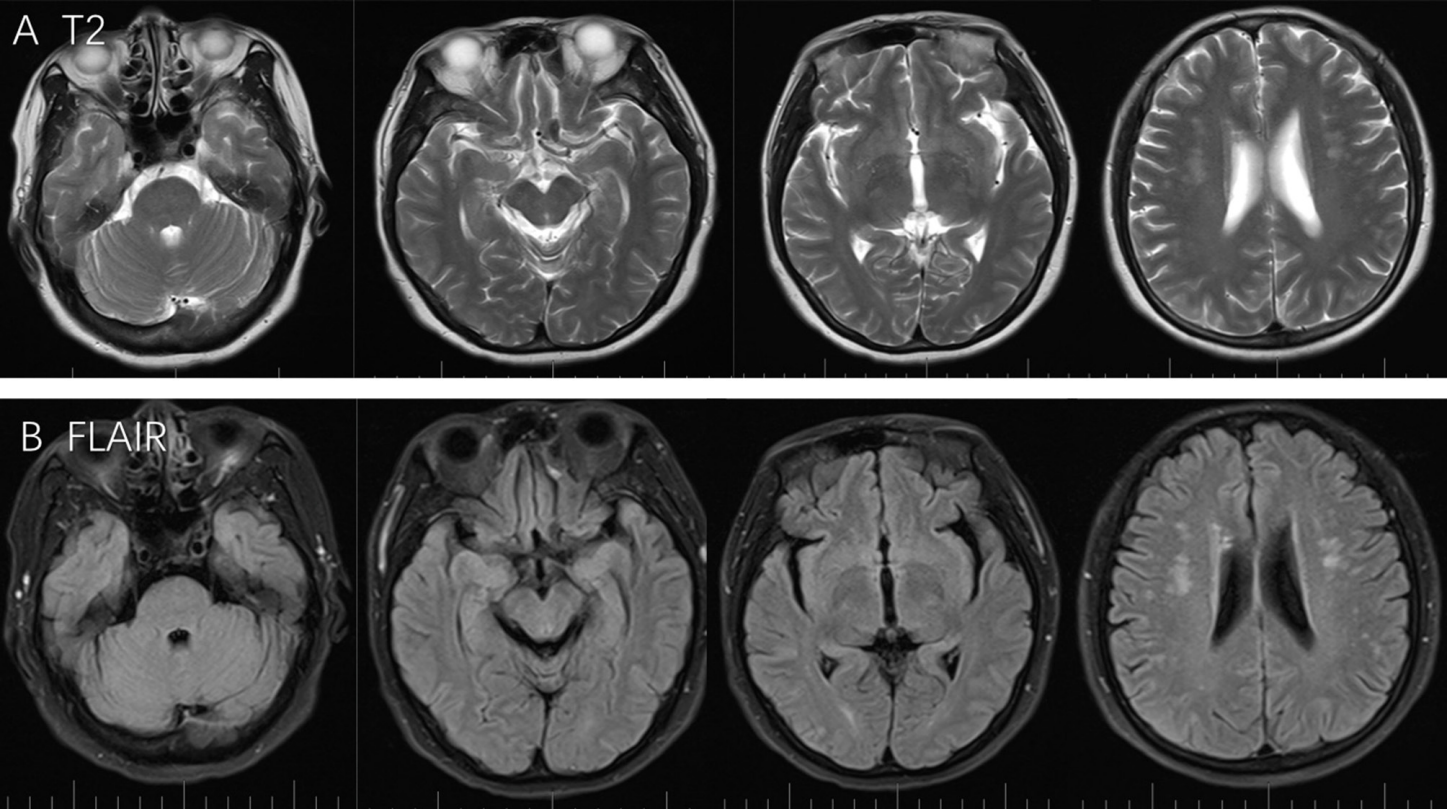
T2 and Flair scans showed full regression from last attack, while no specific lesion was observed around the pons, cerebellum, frontal lobe, parietal lobe, and occipital lobe **(A,B)**.

Three months following her discharge from the hospital, the patient experienced dyspnea due to malignant pleural effusion and was subsequently re-hospitalized. She did not present with any neurological deficits and did not present with hypertension. A brain MRI scan was performed and revealed that the PRES had completely resolved from her previous hospital admission (Figure 2). Further symptomatic treatment was conducted in the oncology department. Graphical abstract of diseases courses is available in Supplementary Data 1.

## Literature Review

Rare adverse effects induced by anlotinib was searched in PubMed between 2018 and 2020, using the terms “Posterior reversible encephalopathy syndrome and anlotinib” and “PRES and anlotinib.” We found six cases of PRES induced by anlotinib in five papers. Clinical details are summarized in Table 1.

**Table 1:** Summary of rare adverse effects induced by anlotinib.

**Case**	**Author/year**	**Age/sex**	**Tumor**	**Treatment of anlotinib**	**History of hypertension**	**Clinical symptoms of AD**	**Diagnosis of AD**	**Reference No**.
1	Jiang B/2019	58/M	Stage IV SqCC	3 months	No	Sudden back pain, sweating and anxiety with increased BP (180/120 mmHg)	Aortic dissection (DeBakey type IIIb)	(6)
2	Liu G/2019	49/M	Stage II SqCC	14 months	No	Chest pain, hyperlipidemia, hand-foot syndrome. and increased BP (140/100 mmHg)	Acute myocardial infarction	(8)
3	Zhang X/2020	48/F	Retroperitoneal leiomyosarcoma with pulmonary metastasis	3 months	No	Loss of vision, severe headache, nausea, vomiting and increased BP (167/113 mmHg)	Hypertensive retinopathy	(10)
4	Li D/2019	69/M	Stage IV NSCLC	42 days	Unknown	Pleuritic chest pain, shortness of breath and nonproductive worsen cough	Bronchopleural fistula	(7)
5	Zhang PL/2019	55/F	Stage IIIb NSCLC	1 month	Unknown	Severe cough after swallowing	Esophago-tracheobronchial fistula	(9)
6	Zhang PL/2019	53/M	Stage IV NSCLC	1 month	Unknown	Severe cough after swallowing	Esophago-tracheobronchial fistula	(9)

*M, male; F, female; SqCC, squamous cell carcinoma; BP, blood pressure; NSCLC, non-small lung cancer*.

All those cases were coming from Chinese patients. Those six cases included four men and two women with the mean age of 55.33 year-old (ranging from 48 to 69 years). All those patients were diagnosed with severe carcinoma reached stage II and above. Adverse effect happened after the mean time of 117 days treatment with anlotinib (ranging from 42 days to 12 months). Those adverse effects include aortic dissection (1 case), acute myocardial infarction (1 case), hypertensive retinopathy (1 case), bronchopleural fistula (1 case), and esophago-tracheobronchial fistula (2 cases). Blood pressure monitoring was performed in first three cases. These three cases, who had no history of hypertension, simultaneously exhibited increased blood pressure during the adverse effects diseases course. The other three cases who diagnosed with bronchopleural fistula and esophago-tracheobronchial fistula had no description about blood pressure. The treatment duration of anlotinib was different in two groups. The former group takes 3–12 months to induce the vascular dysfunction, while the latter group only takes nearly 1 month to induce erosion of adjacent tissue. Although the adverse reactions were treated in time in all six cases, four cases were died of primary disease in the following months, except the case of acute myocardial infarction and hypertension retinopathy.

## Discussion

To the best of our knowledge, we have reported the very first case of PRES induced by Anlotinib. Given the development of novel Cancer immunotherapy has progressed considerably and new techniques for treatment have been introduced (14, 15). The new epoch of highly effective cancer immunotherapeutic options carry risks of probably rare, but severe, adverse effects (16). We believe that this novel case report of anlotinib-induced PRES and review of rare adverse effect have significant implications for cancer patients and prescribing physicians who are seeking to use this novel therapy.

In a phase III clinical trial by China NMPA consisting of 437 patients, anlotinib was found to be attributed to several commonly occurring side-effects including hypertension (67.4%), hand-foot syndrome (43.9%), hemoptysis (14.0%), thyroid stimulation hormone (TSH) elevation (46.6%), and corrected QT interval prolongation (26.2%) (5). Similar adverse effects were reported in a phase II study (3). However, no anlotinib-induced PRES case was reported. While, several studies have found that other anti-VEGF drugs can induce PRES. For example, one study found that anti-VEGF drugs including bevacizumab, sunitinib, sorafenib, aflibercept, pazopanib, and axitinib caused episodes of PRES 9.8-weeks following treatment (17). In alignment with this, these anti-VEGF drugs were also found to be associated with the onset of hypertension (92.3%), headaches (53.8%), seizures (46.1%), and visual impairment (46.1%) (17).

The underlying mechanism of PRES is still unclear. Hypertension is thought to be the main contributing factors, while endothelial dysfunction could play a role in drug-induced PRES, given that 15–20% of patients with normal blood pressure develop PRES (18). Anlotinib, as a VEGF inhibitor could induced endothelial dysfunction in central nervous system, leading to blood brain barrier (BBB) dysfunction and vascular edema. Additionally, hypertension, which is the most commonly seen adverse effect of anlotinib, could also induce PRES. Hence, anlotinib induced endothelial dysfunction and hypertension in our patient, who had no history of hypertension, may both contributed to PRES. Accordingly, in reviewed cases, we found that anlotinib occasionally increase blood pressure in those who had no history of hypertension, which simultaneously happened with vascular involved adverse effects (6, 8, 10). Discontinuation of anlotinib and anti-hypertensive drug could control increased blood pressure. This suggests that BP monitoring and anti-hypertensive drugs may be used to prevent the onset of PRES well before neurological symptoms become apparent with the treatment of anlotinib. It is important to note that anlotinib is metabolized by CYP3A4 (19) and therefore the use of verapamil and diltiazem, which are both CYP3A4 inhibitors, should be avoided.

According to the National Cancer Institute Common Terminology Criteria for Adverse Events version 4.0, the dosage for anlotinib should be reduced to 8–10 mg per day for patients experiencing adverse effects (4, 20). If adverse events continue at 8 mg, then anlotinib should be discontinued. When considering whether a patient may react unfavorably to anlotinib treatment, a study by Liu et al., have shown that decreased CD31-labeled endothelial cells may predict the efficacy of anlotinib treatment. Thus, this may help weigh the risk-benefit of using this drug (21). Moreover, it is important to note that it is feasible to continue treating a patient with anlotinib even after a PRES episode given that the re-administration of anti-VEGF drugs following PRES has been shown not to cause an occurrence of neurological symptoms (22, 23).

The clinical symptoms of PRES are usually sustained for 2–8 days and fully recovered with significant remission as observed on MRI. However, if the pathogenesis is not managed promptly, PRES may induce severe disabilities, including hemiparesis, epilepsy, visual impairment, or death, thereby resulting in permanent neurological disabilities.

## Conclusions

Our case report and review suggest that anlotinib may be damaging to the central nervous system and may cause potentially permanent neurological damage if left untreated. To mitigate this possibility, early prophylactic treatment and dose management should be considered when prescribing anlotinib. Moreover, our study shows that diagnosing anlotinib-induced PRES early may mitigate lasting neurological damage.

## Ethics Statement

Written informed consent was obtained from the individual(s) for the publication of this case report, including any potentially identifiable images or data included in this article.

## Author Contributions

JF and DM conceptualized and designed the study. DN and XY performed literature research, drafted the manuscript. BW helped with the image. XJ revised it critically. All authors have read and approved the final version of the manuscript.

## Conflict of Interest

The authors declare that the research was conducted in the absence of any commercial or financial relationships that could be construed as a potential conflict of interest.

## Supplementary Material

The Graphical Abstract of the Supplementary Material for this article can be found online at: https://www.frontiersin.org/articles/10.3389/fneur.2021.546481/full#supplementary-material
