# Supplementary figures and images for: Posterior Reversible Encephalopathy Syndrome Associated With Anlotinib: A Case Report and Literature Review
Source: Front Neurol. 2021 May 6;12:546481. doi: 10.3389/fneur.2021.546481 (PMC8134686; doi:10.3389/fneur.2021.546481)

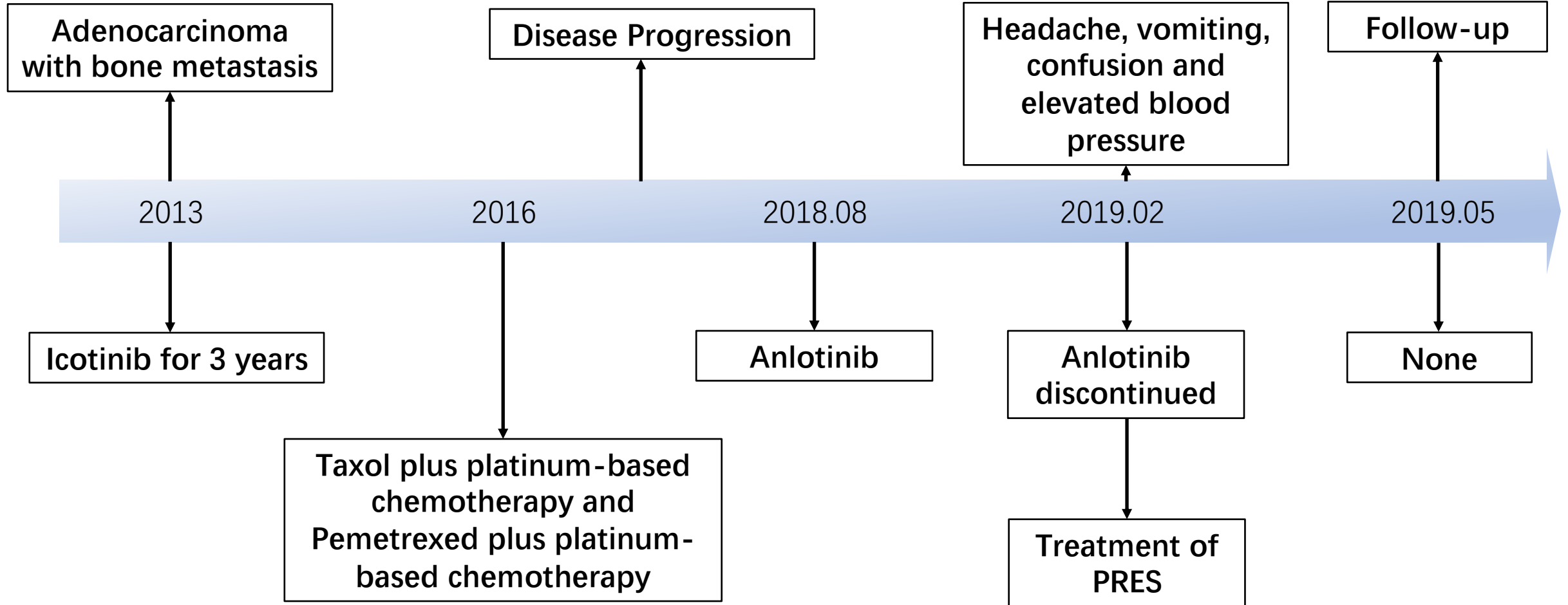

Supplement: Supplementary file 1 [file Data_Sheet_1.PDF]
